# The big food view and human health from the prospect of bio-manufacturing and future food

**DOI:** 10.3389/fnut.2023.1160743

**Published:** 2023-06-08

**Authors:** Jing Wang, Xin Zhang

**Affiliations:** ^1^China Rural Technology Development Center, Beijing, China; ^2^Department of Food Science and Engineering, Ningbo University, Ningbo, China

**Keywords:** big food view, bio-manufacturing, cell factory, future food, sustainable nutrition

## Abstract

The “big food view” has attracted widespread attention due to the view of sustainable nutrition and human health as part of sustainable development. The “big food view” starts from better meeting the people's needs for a better life. While ensuring the supply of grain, the effective supply of meat, vegetables, fruits, aquatic products and other foods also should be guaranteed. Using cell factories to replace the traditional food acquisition methods, establishing a new model of sustainable food manufacturing, will greatly reduce the demand for resources in food production, and improve the controllability of food production and manufacturing, and effectively avoid potential food safety and health risks. Cell factories can provide key technologies and supporting methods for the biological manufacturing of important food components, functional food ingredients and important functional nutritional factors, realizing a safer, nutritious, healthy and sustainable way of food acquisition. The combination of cell factory technology and other technologies meets the people's new dietary demand, and also supports that sustainable nutrition and human health as part of sustainable development. This paper focuses on the big food view and human health from the prospect of bio-manufacturing and future food, which aims to better meet people's dietary needs for increasingly diversified, refined, nutritious and ecological food through diversified food manufacturing.

## Introduction

Recently, the “big food view” has attracted widespread attention due to the view of sustainable nutrition and human health as part of sustainable development (1). The “big food view” is to “start from better meeting the people's needs for a better life.” While ensuring the supply of grain, the effective supply of meat, vegetables, fruits, aquatic products and other foods also should be guaranteed. It includes the structure adjusting and regional layout of the food production, enrich and expand food sources, and actively promote agricultural supply-side reforms based on demand, which fully embodies the concept of “innovation, coordination, green, openness and sharing.”

Healthy, safe, and sustainable food manufacturing is a key element of human health and sustainable social development. The active application of cell factories in the field of food manufacturing has vigorously promoted the rapid development of new foods such as artificial meat, milk, and eggs, becoming an effective way to relieve agricultural pressure and meet the growing food demand (2). As an emerging technology, the use of cell factories to replace traditional food acquisition methods and establish a new sustainable food manufacturing model will greatly reduce the demand for resources in food production, and improve the controllability of food production and manufacturing (3). In the future, this technology is expected to end the use of pesticides and fertilizers, reduce land dependence and pollution, promote the development of agricultural industrialization, and truly realize a new model of efficient, low-carbon and green food production, and expected to become an important guarantee for the “big food view” (4).

This concept is to comply with the changing trend of the people's food consumption structure and ensure that the people eat safe, healthy and nutritionally balanced. In the new era, food consumption has shifted from quantity to quality, and residents are more inclined to buy low-fat, high-protein animal food (5, 6). The increase in the consumption demand of processed food by urban and rural residents is more reflected in the increase of the demand for safe, green and nutritious processed food (7). According to the changing trend of people's consumption, improving the quality and diversity of agricultural products and processed food needs to meet people's demand for food from the supply side and the demand side. On the one hand, the “big food view” requires the government and enterprises to promote the high-quality and differentiated development of agricultural products and food industries, and make efforts from the supply side to meet the people's needs for food diversification, refinement, and nutrition. On the other hand, it requires efforts to promote the renewal of food consumption methods from the demand side (8).

Establishing a “big food view” will subvert the production model of traditional planting and aquaculture, and lead the development of the future food industry (9, 10). Building a “big food view” is inseparable from the innovation of future food technology, which is also a key area for future food production to tackle key problems (11). It requires the use of future food technology, especially food synthetic biotechnology, through factory fermentation production to replace traditional planting and breeding production methods, to make breakthroughs in large-scale, low-cost, sustainable high-efficiency manufacturing of food raw materials such as protein, starch and oil, to achieve “industrialization of agricultural production” (12). For instance, an emerging agricultural method, fish-vegetable symbiosis, uses the symbiosis between fish and plants to uniquely combine hydroponics in a circular aquaculture system with hydroponics in a closed-loop system, in fish-vegetable symbiosis, water is recirculated in a closed loop around the system (13). It also requires future food technology to ensure the high-quality supply of food. For important food ingredients and food functional factors that affect food texture, flavor and nutrition, bio-manufacturing technology is needed to help food precision nutrition and intelligent manufacturing (14). The fourth industrial revolution (Industry 4.0) has revolutionized the way in which food is produced, transported, stored, perceived, and consumed worldwide, leading to the emergence of new food trends (15). The Food Industry 4.0 era has been characterized by new challenges, opportunities, and trends that have reshaped current strategies and prospects for food production and consumption patterns (16).

## The necessity of the concept of “big food view”

The “big food view” needs to be oriented to the “big system,” which includes traditional crops and livestock and poultry resources, as well as special new foods such as artificial milk and artificial meat. To meet this huge demand for food consumption in the world, it is only possible to develop food resources in multiple ways and to develop diverse food varieties.

Quantitative security is always the basis and premise of food security. As people's needs for a better life continue to grow and their expectations for safe and high-quality food grow stronger, food security requires not only a stable increase in the quantity of food, but also a simultaneous improvement in food quality. This requires promoting the transformation of food supply from “quantity-based” to “combining quality and quantity, improving quality and ensuring quantity,” and accelerating the construction of a consumption-driven, rationally structured, and effective food supply guarantee system to better meet the growing consumer demand. On the premise of fully assessing the carrying capacity of resources and the environment and ensuring ecological security in an orderly and reasonable manner, it is necessary to promote the formation of a modern agricultural production structure and regional layout that is in line with market demand and with the carrying capacity of resources and the environment.

The “big food view” is a continuation of the new era of ecological civilization ideas such as “lucid waters and lush mountains are invaluable assets.” Its essence is to achieve the balanced development of ecological protection and agricultural and rural modernization, and to ask for food from mountains, rivers, forests, fields, lakes, grass, and sand on the premise of ensuring ecological security in a reasonable and orderly manner. It is necessary to further broaden the horizons of agricultural talents, and deeply understand the rich connotation of the life community of “landscapes, forests, fields, lakes, grass, and sand” from the perspective of “big food view.” Not just asking for food, but also paying attention to the protection of the ecological environment, unswervingly taking the road of ecological priority and green development, and promoting the integrated protection and systematic management of mountains, rivers, forests, fields, lakes, grass, and sand. Agricultural talents should not only study how to improve water quality, soil quality, and agricultural product quality, but also conduct in-depth research on the living space, habitat space and migration system of animals and plants, so as to achieve a win–win situation in meeting the diverse food needs of the people and protecting the ecological environment, so as to promote more sustainable development.

### The application of “big food view” in emerging technology

Building a “big food view” is inseparable from the innovation of future food technology, which is also a key area for future food production to tackle key problems. Through the supporting role of science and technology, we can search for food from forests and rivers, lakes and seas. At the same time, we can expand biological resources, develop biotechnology and bio-industry, and obtain energy from plant, animal and microorganisms (17).

### Cell factories

In the face of environmental pollution, climate change and increasingly depleted non-renewable resources, how to ensure a healthy, safe and sustainable food supply faces enormous challenges (18). Using cell factory manufacturing to replace traditional food acquisition methods and establishing a new sustainable food manufacturing model will greatly reduce the demand for resources in food production, reduce greenhouse gas emissions, improve the controllability of food production and manufacturing, and effectively avoid potential food production (19). Using cell factories to improve the synthesis efficiency of important food components, functional food additives and nutritional chemicals is an important research direction to solve the current problems faced by food manufacturing.

Cell factory-related technologies provide important technical support for solving the challenges faced by food manufacturing, and are an important research direction in the field of food (20). Remarkable progress has been made in the bio-manufacturing of typical food components represented by key components of plant protein meat and artificial milk (21). The range of target products to be synthesized is to be further expanded to create an “intelligent cell factory” that will significantly improve the efficiency of synthesis of food ingredients and functional foods. Important directions for future research include the realization of whole-cell utilization and industrial-scale preparation.

The cell factory can individually design and adjust products according to requirements, which will be the future development trend of green, safe and high quality food (22). The cell factory will revolutionize the production and supply chain of agricultural products, provide sustainable and healthier food for the growing population, and provide irreplaceable support for the world's agricultural carbon peaking and carbon neutrality goals.

### Artificial milk

Milk proteins are important component in animal milk, which have various biological activities including easy uptake and digestion, high nutrition and immunity enhancement (23). Faced with issues such as sustainability, public health, and animal welfare in dairy production, the research and development of plant- or animal-sourced milk protein replacement technologies has gradually attracted widespread attention (24). Although substantial progress has been made, there are still many problems to be solved in terms of flavor, taste, and functional properties.

Important progress has been made in recent years by using innovative technologies such as synthetic biotechnology to build cell factories with specific synthetic capabilities to produce various agricultural products such as starch, protein, oil, sugar, milk, and meat that humans need (25). The nutrition and flavor of artificial milk are equivalent to natural milk, but it does not contain adverse factors such as lactose, cholesterol, antibiotics and allergens. The production process does not require breeding animals, which can save resources and energy effectively, and will lead the future development of food industry.

### Artificial meat

The overall technical route of artificial food is to build a cell factory to synthesize artificial meat, eggs and milk, etc. by workshop production, so as to relieve agricultural pressure and meet the growing demand for food. Among them, artificial meat is an emerging and breakthrough technology in the food field (Table 1). The breakthrough in large-scale manufacturing technology of artificial meat is expected to reduce the consumption of resources and energy in traditional agriculture (26). In recent years, artificial meat has attracted widespread attention due to its traceability, high food safety and sustainable advantages. Artificial meat can be divided into plant protein meat and cell-cultured meat (27). The plant protein meat has relatively low cost, low technical requirements, and high market acceptance. Therefore, it has the advantages of mature technology and the potential for priority development. A number of companies have developed plant protein products using plant protein as raw materials and have achieved commercial production. Cell-cultured meat is similar to natural meat, but the cost is relatively high in the current stage, and although the market potential is great, it still needs to be fully developed.

**Table 1:** Commercialized artificial food currently developed.

**Time (year)**	**Event**
2009	Beyond Meat Inc. was established in the United States, which provided vegetable meat products
2011	Impossible Foods Inc. was established in the United States, which sold meat free hamburgers and sauces
2019	Mycorena in Sweden produced Fungi-based alternative protein for the food industry
2019	Nature's Fynd in the United States produced microbial protein meat and dairy substitutes
2019	The beef flavor vegetable meat was produced by Jinzi Ham Co., Ltd in P. R. China and Dupont in the United States
2020	KFC launched vegetable meat burgers and chicken nuggets

### Cell-cultured meat

Cell-cultured meat, also called cell-cultivated meat, refers to meat that was produced by livestock and poultry stem cells using tissue engineering technology to produce meat from cell culture (28). In contrast to conventional meat, cell-cultured meat promises to address animal welfare ethics, nutritional properties, and public health issues (29).

At present, cell-cultured meat related products are still mainly in the laboratory research stage, and more comprehensive research and application promotion are needed for widespread market recognition (30). Both plant protein meat and cell-cultured meat need to use key ingredients such as enzymes, vitamins and lipids produced by microbial cell factories (31). The obtained food raw materials and functional ingredients are organically integrated, and finally a recombinant food with a harmonious flavor, stable texture and high simulation is obtained (32).

Cell-cultured meat is commonly expected to alleviate the environmental issues related to the agricultural sector. Nowadays, the livestock operations has used most agricultural land and closely related to the global climate change as well as land and water pollution (33). The production of cell-cultured meat has also been considered to enhance food security, and benefit for the unstable climate conditions (34). The production inside sterile laboratories or factory conditions reduces the concerns of food contamination. Meanwhile, the production of cell-cultured meat prevents the livestock-based infection of epidemics (35). To attract consumers in comparison with conventional meats, the continuous development of cell-cultured meat in sensory and nutritional characteristics is demanded (36).

### Regulation of cell-cultured meat

The regulations on cell-cultured meat are intended to facilitate consumption and investment with a certain sense of safety and assurance under scientific uncertainty. Constraints are possible with naming regarding cell-based meats (37). Laws in the US and EU limit the words including “milk” and “steak” for plant-based alternatives, which will prevent the use of the word “meat” for cell-cultured meat, and it reflects the pressure from traditional livestock organizations.

Keeping the word “meat” to sell cell-cultured meat, make the concept of meat more acceptable to ordinary people. The production of cell-cultured meat demands funds for large-scale production and governments are likely to lead in technical innovation for alternative meats. Governmental policy for R&D can also direct corporations to be greener in developing their products. The spread of alternative meats production can also solve the issues of the work environment in meat processing industries (38).

Although the production of cell-cultured meat may contribute to the resilience of food systems, developing countries without technologies and financial resources for investment may not promote alternative meat production (39). The alternative meat production raises the positive prospect to solve the environmental issues related to current livestock production (40). The urban population in these countries can reasonably depend on alternative meats once the product becomes more common. The attitudes of vegetarians toward cell-cultured meat are another issue. They can eat cell-based meats because they solve the ethical problems about meat production (41).

Recent studies have discussed the impact of cell-cultured meat production on environmental factors, such as greenhouse gases emissions, land use, energy use, and water use (42). The production of cell-cultured meat demands much fewer resources than European beef, and the greenhouse gases emission was more than 78% less (21). The technological development of cell-cultured meat can target the entire process of production to consumption including texture refinement and composition improvement (43). Furthermore, the policies must integrate social, ethical, and environmental concerns with energy-efficient production and connect with current efforts on carbon peaking and carbon neutrality goals and biodiversity conservation. The production of cell-based meats continuously demands sound science-policy discussion to resolve the contestation over cell-based meats, which will form the basis for current R&D activities and future industrialization.

However, there is still a big gap between the quality of artificial meat and real meat, and breakthroughs in texture simulation, nutritional optimization, flavor adjustment, and product customization are urgently needed. Using enzymes with specific functions, can achieve structural strength modification, structural hydrophilicity and hydrophobicity modification, allergen degradation, protein utilization improvement, and glycolipid protein integration, degradation of odor components and enhancement of flavor substances. The synergistic utilization of enzymes and flavor substances with specific functions will improve the quality of artificial meat, and lay the foundation for the customized process of finished products based on 3D printing food.

### 3D printing

Three-dimensional (3D) food printing technology combines 3D printing and food manufacturing. The potential of delivering personalized products tailored to meet the taste preferences and specific dietary needs is one of the reasons for increasing researches in this technology (44). With the increased living standards, people have higher demand on healthy functional food and even personalized food (45).

Food 3D printing can be blended with various raw materials according to individual's physical and nutritional status, so that the functional factors such as protein, fat, dietary fiber, vitamins, and minerals, are balanced according to the demand (46). In food processing, and realize digital nutrition and complex food design, which cannot be achieved by traditional food processing methods (47).

The 3D food printing combines 3D printing technology and food manufacturing and uses edible materials such as fruit and vegetable juice and powder, starch, meat, chocolate, and algae etc. as printing materials (48). As we know, the most important feature/advantage of 3D printing is the creation of complex 3D structures. But in food field, the potential of delivering personalized nutrition and personalized food choice may be the main reasons that the 3D food printing technology is advancing so rapidly. 3D food printing technology can enable formulation of food to meet the need of people having different preferences for taste, dietary needs and physical condition such as dysphagia (49). Specifically, tailored foods by adding specific nutrients and functional compounds or eliminating/replacing certain ingredients in the formulation can help promote health and prevent diseases (50).

The 3D printing technology can seamlessly integrate nutrition, enable manufacturing of personalized foods that satisfy the requirements of consumers according to their occupation, gender, age, and lifestyle (51). Food with desirable texture has been produced using 3D printing by developing different nozzles and filling modes (52). The 3D printed foods can be popular and more appealingly designed to cater for specific needs of the children (53). 3D printing technology helps to create healthy snacks with novel shapes and rich in vitamins and minerals, attracting children and becoming a model of personalized food. In fact, 3D printing enables manufacturing of personalized foods that have both health promoting and enjoyment elements (54). 3D printing is an innovation that promises to revolutionize food formulation and manufacturing processes. Preparing foods with customized sensory attributes from different ingredients and additives has always been a need (55).

The future task of the food industry is to focus on developing robust 3D food printers, understand material printability, identify unique food sources for printing, and simplify the technology for convenient use by all. An added aspect is to focus on the effect of pre- and post-printing operations on the quality and consumer acceptance of 3D-printed foods, and overall process control. While pre-processing involves alterations in material supply ingredients to make a recipe compatible for printing, post-processing of fabricated structures focuses on improving the palatability of 3D-printed foods.

## The significance and value of future food

The manufacturing mode of the traditional food industry may be changed in the future. It is mainly through the combination of food and biotechnology to change the traditional methods of cultivation and reproduction and production. The typical representative is artificial meat, including plant protein meat made from soybean and other plant protein as raw materials, and cell-culture meat made from cells extracted from animals.

Food will make people and the earth healthier. The chronic diseases are caused by the way people eat and drink. A large number of medical studies showed that adding certain plant protein to animal protein can significantly reduce the risk of death (56, 57). Now, the way of livestock and poultry breeding to obtain animal protein is much higher than that of plants, microorganisms to obtain protein, in terms of resource occupation and environmental impact. Substitute protein not only has the above resource and environmental benefits, but also has obvious advantages over traditional livestock and poultry breeding in terms of protein production efficiency.

The core content of future food includes plant-based food, alternative protein, food perception, etc. The development of food in the future will highlight six “new”: breakthroughs in food nutrition and health will become the new engine of food development; the progress of physical properties of foods will become a new source of food manufacturing; the results of the detection and control of food hazards will become a new support for active security; the innovation of green manufacturing technology will become a new driving force for the sustainable development of the food industry; the revolution of intelligent equipment for food processing will become a new driving force for the upgrading of the food industry; the integration of the whole food chain technology will become a new model of the food industry.

## Conclusion

It is the starting point of the “big of food view” to improve food production, improve quality and diversity through scientific and technological innovation, at the same time open up new food access so that people can have enough, good and healthy food and better satisfy people who yearn for a better life. Since the beginning of the new century, the emerging science such as synthetic biology, bioinformatics, bionics and artificial intelligence have developed rapidly, which is promoting the transformation of agricultural production mode from traditional agriculture and industrial agriculture to cell agriculture. Cell factory is one of the main methods to solve the major challenges of food in the future, including the development and high-value utilization of new food resources and the transformation of diversified food production mode. Cell factories can personalized design and adjust products according to requirements, which will be the future development trend of green, safe and high-quality food. We should strengthen the research and development of cell factory-related technologies, and meet people's new dietary needs through diversified food manufacturing ways.

## Author contributions

JW: conceptualization and writing original draft. XZ: supervision and writing review and editing. Both authors contributed to the article and approved the submitted version.
